# Remodeling of RecG Helicase at the DNA Replication Fork by SSB Protein

**DOI:** 10.1038/srep09625

**Published:** 2015-04-29

**Authors:** Zhiqiang Sun, Hui Yin Tan, Piero R. Bianco, Yuri L. Lyubchenko

**Affiliations:** 1 Department of Pharmaceutical Sciences, University of Nebraska Medical Center, Omaha, NE 68198-6025, USA; 2 Department of Microbiology and Immunology, University at Buffalo, SUNY, Buffalo, NY 14214, USA

## Abstract

The RecG DNA helicase a key player in stalled replication fork rescue. The single-stranded DNA binding protein (SSB) participates in this process, but its role in the interaction of RecG with the fork remains unclear. We used atomic force microscopy (AFM) to visualize the interaction of RecG with a fork DNA in the presence of SSB. We discovered that SSB enhances RecG loading efficiency onto the DNA fork by threefold. Additionally, SSB interacts with RecG leading to the RecG remodeling. As a result, RecG separates from the fork, but remains bound to the DNA duplex. Moreover, in this new binding mode RecG is capable of translocation along the parental duplex DNA. We propose a model of RecG interaction with the replication fork involving two RecG binding modes. SSB plays the role of a remodeling factor defining the mode of RecG binding to the fork mediated by the SSB C-terminus. In the translocating mode, RecG remains in the vicinity of the fork and is capable of initiating the fork regression. Our results afford novel mechanistic insights into RecG interaction with the replication fork and provide the basis for further structural studies.

Genome duplication is an inherently accurate and highly processive process that relies on the close interplay between genetic recombination and DNA repair machinery^1^,^2^,^3^. This interplay arises because the replication machinery frequently encounters roadblocks that have the potential to stall or collapse a replication fork^4^,^5^,^6^. The stalled fork can be reversed (regressed) through a process catalyzed by the RecG helicase in an ATP-dependent manner^7^,^8^, leading to the formation of a Holliday Junction-type (HJ) structure that is then processed by RuvAB^7^,^9^. Crystallographic data for RecG in complex with fork DNA led to a model in which one of the RecG domains interacts with the replication fork, while two other domains bind to the parental DNA duplex^10^. The authors also propose a model for ATP-dependent fork regression in which the coordinated function of all RecG domains allows the protein to move along the parental DNA duplex, causing the nascent leading and lagging DNA duplexes to unwind, leading to the formation of the HJ structure. However, in this mode, RecG works against the fork movement, suggesting that RecG should be in inactive state when replication fork progresses. However, the mechanism of RecG inactivation remains unknown. Additionally, recent studies suggest that the activity of RecG in the early stages of fork rescue is enhanced and controlled by SSB^8^,^9^,^11^,^12^, but the mechanism of this interaction remains unclear.

It was widely accepted that the role of SSB in DNA metabolism is primarily limited to its specific binding to single-stranded (ssDNA) to prevent DNA strand annealing or/and ssDNA degradation^13^,^14^,^15^. More recently, it has become clear that SSB binds to a group of at least twelve proteins known as the “SSB-interactome” that includes the DNA helicases PriA and RecG, exonuclease I, components of the DNA replication machinery, and topoisomerase III^16^. SSB binding facilitates the actions of these interactome proteins, but how this occurs is unknown. It is also unknown how RecG specifically targets a fork within the milieu of the genome and initiates fork rescue.

Here we clarified both mechanisms using atomic force microscopy (AFM) to visualize the interaction of RecG with a fork DNA in the presence of SSB. We discovered that RecG is capable of binding to the fork in two different modes and SSB is involved in this conformation switch enhancing RecG loading efficiency and remodeling the helicase. As a result, remodeled RecG separates from the fork, but remains bound to the parental DNA duplex arm. In such new binding mode, RecG can move freely along the parental arm, but remains in the fork proximity being available for the fork regression.

## Results and Discussion

### Experimental design

**Figure 1:**
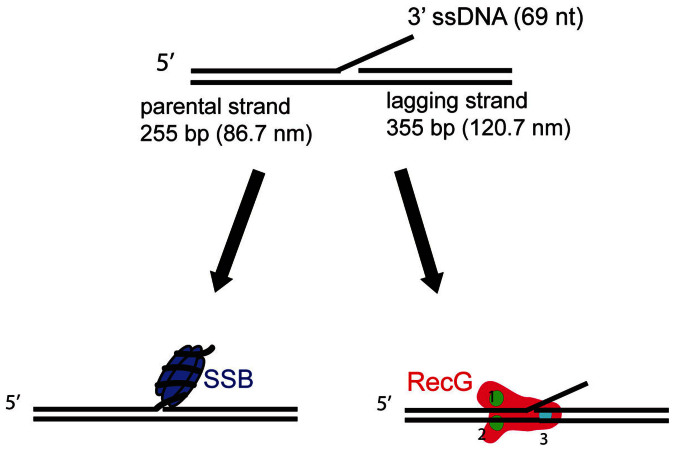
Fork DNA structure and anticipated interactions with SSB and RecG. The fork DNA contains a 69 nt 3′-end ssDNA tail, 255 bp dsDNA on parental strand and 355 bp dsDNA on lagging strand. The fork DNA construct was assembled as shown in Fig. S1. SSB is shown bound to the ssDNA arm of the fork. According to Ref. 10, RecG binds to the fork specially using its three domains.

A schematic of the fork DNA template and anticipated interactions with SSB and RecG is shown in Fig. 1. The DNA substrate is a fork with a gap in the nascent leading strand, the preferred substrate for RecG^10^,^17^, it contains a 3′-end 69 nt single-stranded DNA (ssDNA) segment inserted between two DNA duplexes of different lengths (255 and 355 bp). Figure S1 provides specifics for the fork design and assembly. This type of construct mimics a stalled fork with a ssDNA gap on the nascent leading strand and is generally similar to the substrate used for crystallographic studies of RecG-fork DNA complexes^10^. According to the crystallographic model, RecG domains 1 and 2 form a complex with the parental duplex DNA ahead of the fork, while domain 3 (the wedge domain) interacts with the ssDNA segment of the fork. With such a design we should be able to unambiguously identify the interactions of both proteins with the fork.

### Interaction of SSB and RecG separately with the fork substrate

**Figure 2:**
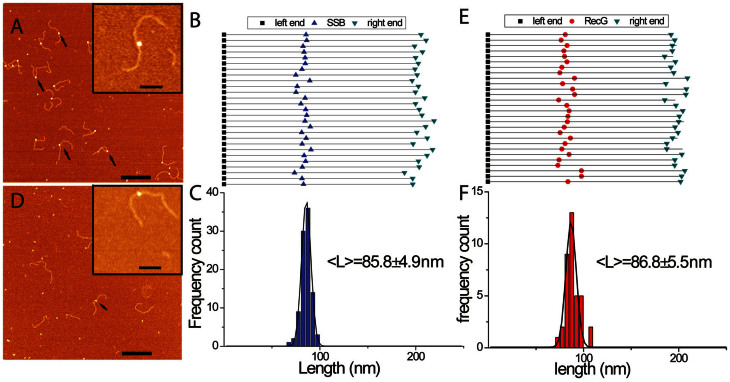
AFM analysis of the interaction of SSB and RecG with the fork DNA substrate. (A) and (D) show representative AFM images of SSB and RecG only to the fork DNA. Arrows point to the complexes. Bar size is 200 nm. Insets show enlarged images of complexes. Bar size 50 nm. (B) and (E) show maps for positions of SSB and RecG, respectively, on the DNA substrate. The DNA molecules were aligned by the left end of the parental flank without normalization of the molecules lengths. (C) and (F) are the distributions of the proteins distances from the left end for SSB (n = 95) and RecG (n = 37), respectively. The distributions were fitted by Gaussians and the maxima values ± SD are indicated on the histograms.

A typical AFM image of the SSB-fork complex is shown in Fig. 2A in which SSB appears as a bright feature located on of the DNA substrate. The locations of SSB on the DNA molecules were mapped, and the results in Fig. 2B and C directly illustrate the correlated position of the proteins on different DNA molecules. Statistical analysis of DNA length measurements shows a narrow distribution for the SSB positions, with a mean value of 85.8 ± 4.9 nm, corresponding to the expected position of the fork within the DNA substrate (86.7 nm, Fig. 1). The yield of complexes is 85.3 ± 3.8%, similar to our recent results of SSB-DNA complexes obtained for the tail DNA substrate^18^. Importantly, SSB does not bind to duplex DNA under these reaction conditions. These data demonstrate that SSB binds specifically to the ssDNA arm of the fork, which is also in line with our previous findings^18^.

Similar experiments were performed with RecG. In the AFM image shown in Figure 2D, RecG appeared as bright particles on the DNA indicated by arrows. Even though this is the preferred substrate for RecG, the yield of protein-DNA complexes was only 10%. This is significantly lower than SSB, although a two-fold higher concentration of RecG was used. RecG's DNA binding sites were mapped (Fig. 2E and F). The mean value for the lengths of the left flank of the DNA molecules was 86.8 ± 5.5 nm, corresponding to the position of the fork in the DNA substrate (86.7 nm). Further evidence demonstrating fork-specific binding by RecG comes from control experiments using a 3′-tailed DNA molecule (Supplementary Fig. S2A). SSB binds to this substrate with an efficiency similar to that of the fork DNA molecule, whereas we were unable to detect RecG binding to 3′-tail-DNA. Therefore, RecG binds specifically to the fork, but binding is much less efficient than that of SSB.

A visual comparison of the images for fork DNA-protein complexes shows that the brightness of the SSB molecules is higher than those of the RecG molecules (Fig. 2D). This was confirmed by the height and volume measurements for the two proteins (Supplementary Fig. S3). The mean values for the volumes and heights for SSB were 122.8 ± 22.1 nm^3^ and 3.43 ± 0.22 nm, respectively. The same parameters for RecG were 44.5 ± 11.7 nm^3^ and 1.51 ± 0.16 nm, respectively. Similar size differences between free SSB and RecG proteins were also observed (Supplementary Fig. S4). These differences in height and volume become critical in distinguishing SSB from RecG when bound to the same DNA molecule, as described below.

### Interaction of SSB and RecG together with the fork substrate

**Figure 3:**
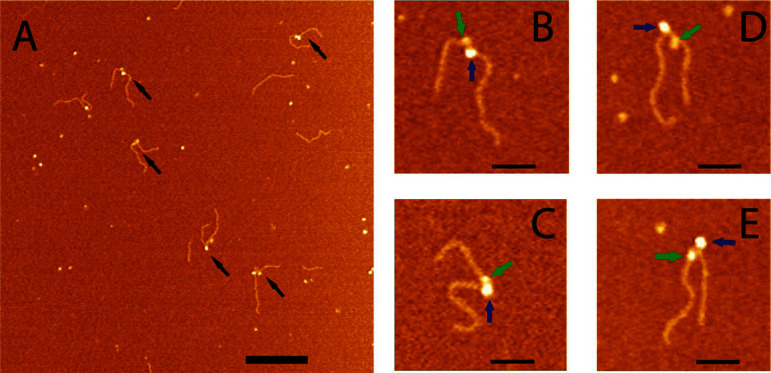
AFM images of the complexes made by SSB and RecG with the fork DNA substrate. (A) Large scale AFM images, in which double-particle features are indicated with arrows. Bar size is 200 nm. Zoomed images (bar size 50 nm) of four double-particle complexes are shown in plates (i) to (iv). Large and small particles are indicated with blue and green arrows, respectively. DNA was first mixed with SSB in the molar ratio of 1:2 for 10 min, then RecG in a ratio of 1:4 was added, and the incubation was continued for 30 min at room temperature. The black arrows point to the double-particle complexes.

Next, we performed experiments in which both SSB and RecG were bound to the same DNA substrate. In these experiments, SSB was bound first, then RecG was added, and the mixture was incubated at room temperature for 30 min, followed by AFM imaging. A typical AFM image is shown in Fig. 3A. The new feature in this image is the appearance of complexes containing two particles, as indicated by arrows. Four zoomed images of double-particle complexes are shown in plates B–F, in which two particles with different sizes are indicated with green and blue arrows. The volumes and heights of the particles in the double- particle complexes were measured and fitted by Gaussians (Supplementary Fig. S5A and C). The heights for the large and small particles were 3.34 ± 0.48 and 1.54 ± 0.19 nm, respectively. These numbers are very close to the values obtained for the individual SSB-DNA and RecG-DNA complexes. The same correlation was obtained for the volume measurements; therefore, the large and small particles are SSB and RecG, respectively. These data demonstrate that RecG is capable of binding to the DNA fork in the presence of SSB, because the two proteins appear on the same DNA substrate simultaneously.

Along with double- particle complexes, single-particle complexes were also observed in the samples. These could be SSB-RecG complexes with the two proteins tightly associated. Therefore, we measured the sizes of single-particle complexes to determine their identity. The mean height was 3.47 ± 0.21 nm, and the mean volume was 124.2 ± 25.8 nm^3^ (Supplementary Fig. S5B and D). These numbers coincide with sizes of complexes for SSB alone. Additionally, the total yield of these single-particle complexes is the same as the yield of SSB-DNA complexes only. Altogether, these data suggest that the single-particle complexes in this sample contain SSB protein only. As stoichiometric ratios of SSB to RecG were used in these experiments, we conclude that these proteins do not remain stably associated with each other in the complexes with the fork DNA substrate.

### SSB facilitates the RecG binding to the fork

**Figure 4:**
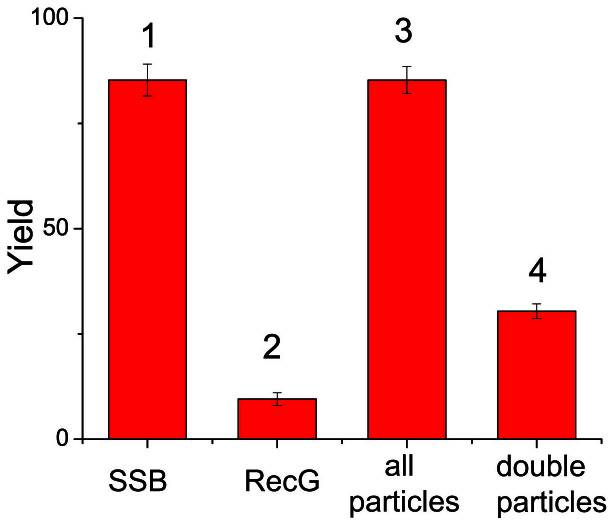
Yield of complexes depending on the sample compositions. Columns 1 and 2 correspond to the yield of complexes with SSB or RecG only. Columns 3 and 4 correspond to the yield of all complexes and double-particle features, in samples containing DNA with both SSB and RecG.

As it is evident form the AFM images, the number of RecG-DNA complexes appearing in the AFM images as double-particle complexes is considerably larger than those for RecG-DNA complexes only (cf. Fig. 3A and 2D). The direct measurement of the yield of double- particle complexes is ~30.3%, while the yield of RecG-DNA complexes is 9.7%, as shown in the histograms in Fig. 4, bars 2 and 4. This finding suggests that SSB facilitates RecG binding to the fork DNA substrate. We performed similar experiments with the tail-DNA substrate that has the same ssDNA at the end of the duplex. In these control experiments, SSB binds specifically to the end of the DNA substrate (Supplementary Fig. S2A), but RecG does not appear on this DNA substrate, regardless of the presence or absence of SSB (Supplementary Figs. S2B and C). Thus, SSB facilitates RecG loading onto the fork DNA only.

**Figure 5:**
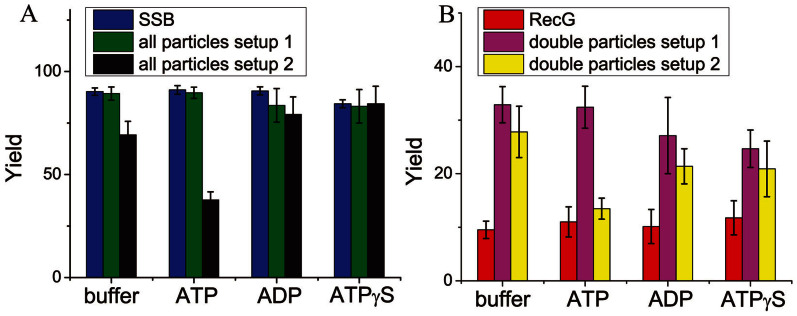
The yields of SSB or RecG-DNA complexes prepared in different buffers. (A) The yield of SSB-DNA complexes either for SSB complexes with the DNA (blue bar), or SSB-DNA in the double-molecule complexes obtained in the presence of both proteins. The green bar corresponds to the complex formation formed by setup 1, in which DNA was mixed with SSB and RecG was added later. The black bar shows the SSB-DNA yield for setup 2, in which both proteins were preincubated and then DNA was added. The first set of columns, denoted by “buffer”, corresponds to the regular conditions, and the other data sets correspond to the same buffer in the presence of 1 mM ATP or ADP or ATP-γ-S, denoted by “ATP”, “ADP”, and “ATP-γ-S”. (B) The yields of RecG-DNA complexes obtained for the complex of fork DNA with RecG only (red column), and for the double-molecule complexes obtained in setup 1 (pink column) and setup 2 (yellow column). Buffer conditions for these experiments were prepared in the same manner to those used for the data presented in (A). Number of complexes analyzed varied between 143 and 234.

**Figure 6:**
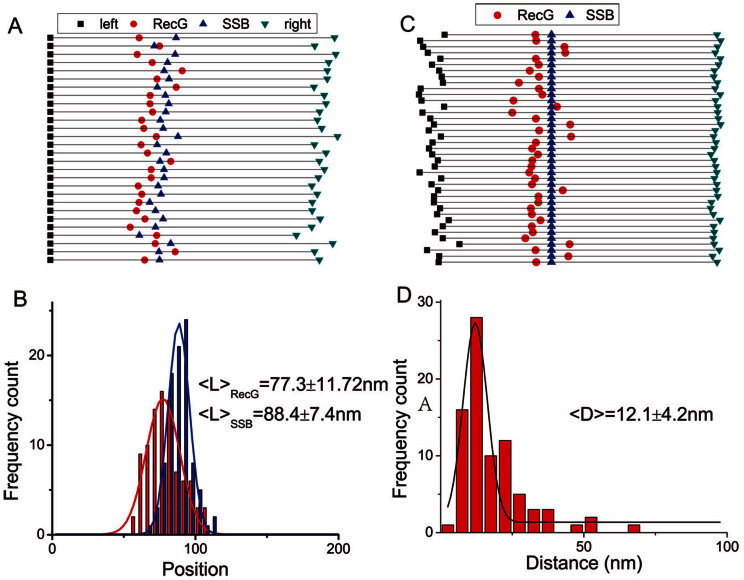
AFM analysis of the RecG and SSB location on the DNA substrate. (A) Linear maps taken from AFM images, similar to those in Figure 3A, with the positions of RecG indicated by red circles and SSB indicated by blue triangles. The DNA molecules were aligned to their left ends. (B) Histograms for the distances between the positions of RecG and SSB relative to the left end. The distributions (n = 91) are approximated with Gaussians, and the mean values ± SD are indicated. (C) Maps of the RecG positions (red circles) relative to SSB bound to the same DNA. (D) Distance distributions between SSB and RecG are shown in the histogram. The distribution (n = 91) is approximated with Gaussians, and the mean value ± SD is indicated.

We also tested whether preincubation of the proteins before binding to the fork affects the efficiency of RecG loading. We premixed SSB and RecG for 30 min, then added the mixture to the DNA substrate, incubated for 30 min, and imaged with AFM. Typical AFM images of these complexes are shown in Supplementary Fig. S6B. The double-particle complexes also appear following preincubation of SSB and RecG, as indicated with arrows. Statistical analysis (Fig. 5B) showed that the yield of double-particle complexes in both experimental designs is very close, 33% for SSB preincubation and 27% for SSB-RecG preincubation (Fig. 6B). Therefore, we conclude that SSB facilitates RecG loading onto the DNA fork regardless of the complex preparation method.

### SSB remodels RecG in the complex with the fork

We measured the positions of both proteins on the DNA in double-particle complexes. The maps are shown in Fig. 6A, with RecG represented by red circles, and SSB represented by blue triangles. Histograms in Fig. 6B, built based on the mapping results, show the positions of RecG (red) and SSB (blue) relative to the left end of the DNA substrate. Unexpectedly, the protein positions do not coincide. The SSB position is 88.4 ± 7.4 nm, which is very close to the SSB alone position (Fig. 2C). In contrast, the position of RecG is shifted toward the left end of the DNA substrate, and the maximum of the distribution is 77.3 ± 11.7 nm, while the position of RecG alone is 85.8 ± 4.9 nm (Fig. 2F). These data suggest that after RecG binds to the DNA fork, it does not remain at the fork. Instead, it translocates along the parental DNA arm ahead of the fork. The translocation activity was not observed when the RecG-DNA complexes were assembled without SSB, suggesting that interaction with SSB is required to allow RecG to translocate along the duplex DNA.

In order to characterize the RecG translocation, we mapped the RecG positions relative to SSB in the double-particle complexes, with the SSB position remaining unchanged relative to the fork position. The mapping results, shown in Fig. 6C, demonstrate that RecG is primarily localized on the parental DNA segment of the fork (79%). The histogram for the inter-protein distances is shown in Fig. 6D. The maximum of this distribution is 12.1 nm, which corresponds to translocation distances as large as ~35 bp, although several larger distances are identified as well. Therefore, we assume that SSB facilitates efficient RecG-DNA complex formation, and remodels the RecG-DNA fork complex, allowing RecG to translocate along the DNA duplex.

As RecG is an ATP-dependent helicase^8^, we wanted to determine if ATP hydrolysis was critical to SSB-induced translocation by RecG. Surprisingly, results show that neither nucleoside binding nor hydrolysis is required (Fig. 5A, B). Consequently, this translocation is thermally driven.

**Figure 7:**
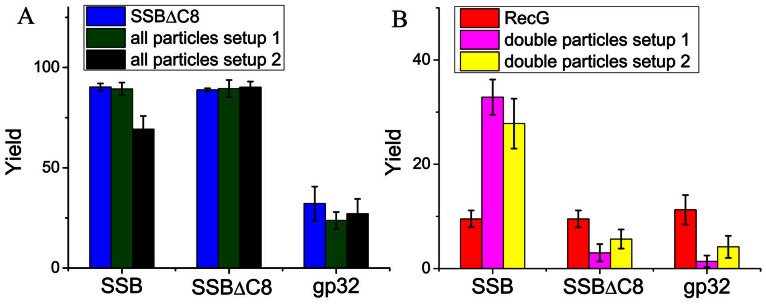
The yields of protein-DNA complexes for different single-stranded DNA binding proteins. (A) The yield of SSBΔC8 or gp32-DNA complexes (containing all molecules), depicted according to the experimental setup. The blue columns show only SSBΔC88 or gp32 mixed with DNA. The green and black columns show the yields of SSBΔC8 or gp32 complexes, in the presence of RecG for setups 1 and 2 (see Fig. 5 for specifics). (B) The yield of RecG-DNA complexes, dependent on the presence of SSBΔC8 or gp32. The pink and yellow bars correspond to the yield calculated from the double-molecule complexes. The red columns show the yield when RecG only was mixed with DNA. The conditions used for the experiments represented by the pink and yellow columns are the same as those used in (A). Number of complexes analyzed varied between 146 and 244.

Additionally, we compared the effect of full-length SSB to SSBΔC8, in which eight residues of unstructured C-terminus were deleted. AFM images in Supplementary Fig. S6D and the histograms (Fig. 7A) demonstrate that SSBΔC8 binds to DNA with an efficiency similar to that of wild type, but does not facilitate RecG binding to the fork DNA (Fig. 7B). Furthermore, in the presence of SSBΔC8, the yield of RecG-DNA complexes was even lower than RecG only experiments, consistent with previous data showing that this mutant form of SSB negatively affects RecG^9^. Similar experiments were performed with the T4 gp32 protein, another ssDNA binding protein. Figures 7A and B show that gp32 binds to the fork less efficiently than SSB, with a yield of 24% for gp32 versus 85.3% for SSB. It also decreases RecG loading efficiency onto the fork (Supplementary Figs. S6F and 7B). Therefore, loading and remodeling of RecG is SSB specific and requires the C-terminus of SSB.

### Two modes of RecG binding to the fork DNA

**Figure 8:**
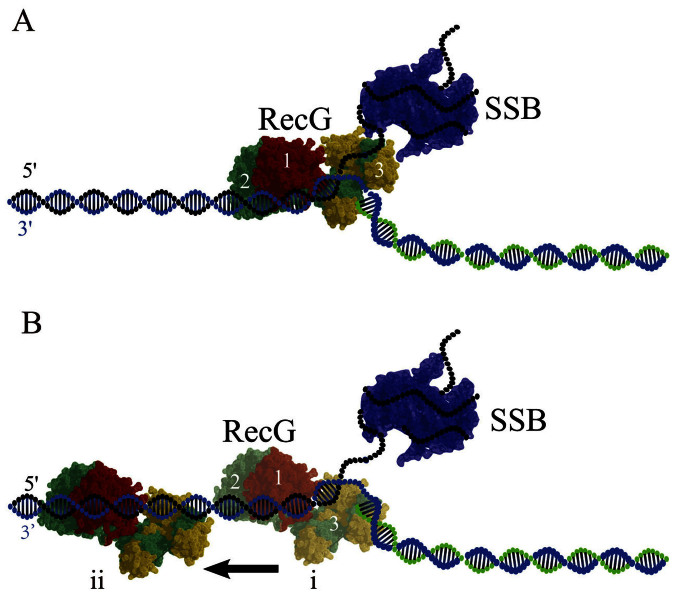
Models for RecG binding to the DNA fork in the presence of SSB. (A) – no interaction with SSB. In type 1 complex RecG binds specifically to all arms of the fork in which domains 1 and 2 bind the parental DNA duplex, whereas wedge domain 3 binds single-stranded arm of the fork. SSB occupies the ssDNA arm of the fork. (B) In type 2 complex formed after the SSB remodeling, the wedge domain 3 dissociates from the ssDNA arm, but domains 1 and 2 remain bound to the parental DNA flank, enabling RecG to translocate along the duplex as shown in (i) and (ii). Model (i) shows the RecG position at the fork just after re-modeling and (ii) shows RecG position after translocation away from the fork.

On the basis of the crystallographic model for the RecG-fork DNA complex, we propose a model for SSB mediated RecG remodeling (Fig. 8). In the absence of interaction with SSB according to the crystallographic model^10^, RecG binds to the fork using all three domains and interaction of the wedge domain with the single-stranded arm of the fork provide specific binding of RecG to the fork (Fig. 8A). In contrast, RecG interaction with SSB leads to the RecG remodeling in which the wedge domain disengages from the fork, but domains 1 and 2 remain bound to the DNA duplex (Fig. 8B (i)). As a result, RecG loses the “hook” that kept it bound to the fork, allowing the protein to translocate along the duplex as it is shown schematically by Fig.8B (ii). The mean migration range is as large as ~35 bp, suggesting that RecG in this binding mode is capable of scanning a rather large DNA segment. Importantly, RecG remains in the fork vicinity and can be recruited for the fork regression when the fork stalls. We hypothesize that this remodeling of RecG switches off its the helicase activity maintaining RecG in the proximity of the replication fork. Note that the fork regression requires ATP hydrolysis^19^, whereas the translocation by the remodeled RecG is a thermally driven process and does not require ATP. Therefore, RecG can be recruited rapidly to accomplish its fork regression role.

Although SSB is considered as architectural single-stranded DNA binding protein with no sequence specificity for binding DNA, evidence accumulated recently point to interaction of SSB with more than a dozen proteins^15^. The experiments described here demonstrate a novel role of SSB in which the protein dramatically increases RecG loading efficiency onto the DNA fork. Moreover, SSB plays a role of a remodeling factor of RecG after which the helicase disengages from the fork but remains bound to the parental duplex. Importantly, the C-terminus of SSB plays a critical role for both new SSB activities and this finding is in line with early findings of the role of this region of SSB interaction with other proteins^15^. Interestingly, that SSB and RecG do not form stable complexes that could be detected by AFM suggesting that the interaction is transient. Given the fact that SSB is involved in other genetic processes, it is reasonable to assume an active role of SSB is not be limited to the DNA replication process, but can be found in other processes requiring SSB. Much work remains to test this hypothesis and to establish the mechanistic bases for SSB role in the replication and other genetic processes. Our study provides a first step toward this mechanistic understanding.

## Methods

### Protein preparation

*RecG* protein was purified as described previously^8^,^20^. The first column was a 30 ml Q-Sepharose column equilibrated in buffer A [20 mM Tris–HCl (pH 8.5), 1 mM EDTA, 1 mM DTT, 10 mM NaCl]. The protein was eluted using a linear gradient (10–1000 mM NaCl), and RecG eluted between 250 and 360 mM NaCl. The pooled fractions were subjected to heparin FF and hydroxylapatite chromatography. Pooled fractions from the hydroxylapatite column were dialyzed overnight into S buffer [10 mM KPO_4_ (pH 6.8), 1 mM DTT, 1 mM EDTA and 100 mM KCl]. The protein was applied to a 1 ml MonoS column and eluted using a linear KCl gradient (100–700 mM), with RecG eluting at 350 mM KCl. The fractions containing RecG were pooled and dialyzed overnight against storage buffer [20 mM Tris–HCl (pH 7.5), 1 mM EDTA, 1 mM DTT, 100 mM NaCl and 50% (v/v) glycerol]. The protein concentration was determined spectrophotometrically using an extinction coefficient of 49 500 M^−1^ cm^−1^.

SSB proteins: *Escherichia coli* single-stranded DNA binding protein (SSB) was purified from strain K12ΔH1Δtrp, as described previously^21^. The concentration of purified SSB protein was determined at 280 nm using ε = 30 000 M^−1^ cm^−1^. The site size of SSB protein was determined to be 10 nucleotides per monomer by monitoring the quenching of the intrinsic fluorescence of SSB that occurs by binding to ssDNA, as described previously^22^. The his-SSBΔC8 mutant protein was purified using nickel column chromatography, followed by step elution from ssDNA-cellulose, similar to wild type. Bacteriophage gene 32 protein (gp32) was purified as described in^16^. The concentration of purified gp32 was determined at 280 nm using ε = 37 000 M^−1^ cm^−1^. The site size of gp32 was determined to be 7 nucleotides per monomer by monitoring the quenching of the intrinsic fluorescence of gp32 that occurs by binding to ssDNA.

### Preparation of DNA-protein complexes

1. SSB-DNA complex. SSB tetramer (0.5µl, 50 nM) was mixed with the fork DNA substrate (0.5µl, 24 nM) at the 2:1 protein to DNA molecular ratio in 10µl buffer containing 10 mM Tris-HCl (pH 7.5), 50 mM NaCl, and 5 mM MgCl2, 1 mM DTT, incubated for 10 min at room temperature.

2. RecG-DNA complex. RecG protein (1µl, 50 nM) to DNA (0.5µl, 24 nM) molar ratio was 4:1 and the mixture was incubated for 30 min in 10µl same buffer as that of SSB-DNA complex.

3. Complex of DNA with two proteins. Two approaches were used. In one approach, setup1, SSB tetramer (0.5µl, 50 nM) was mixed with DNA (0.5µl, 24 nM) in 2:1 molar ratio in 5µl buffer for 10 min, and then RecG (1µl, 50 nM) was added to the mixture in 4:1 RecG-to-DNA ratio in final 10µl buffer and incubated for 30 min. In the second approach, setup2, SSB (5µl, 50 nM) was premixed with RecG (10µl, 50 nM) in 1:2 molar ratio and incubated for 30 min on ice, then DNA (0.5µl, 24 nM) was mixed with the protein mixture (1.5µl) in 10µl buffer and the reaction was continued for additional 30 min at room temperature. Similar protocols were applied to experiments with SSBΔC and gp32 instead of SSB in the reactions. In the experiments with the use of ATP, ADP and ATP-γ-S, 1 mM these chemicals were added to the buffer.

### Dry sample preparation and AFM imaging

The APS mica procedure was used, in which freshly cleaved mica is treated with 1-(3-aminopropyl)silatrane (APS), as described in^18^,^23^. Five microliters of the sample was deposited on APS mica for 2 min, rinsed with deionized water, and dried with Argon gas. Images were acquired in air in Peak Force mode, using the Multimode Nanoscope VIII system (Bruker, Santa Barbara, CA) and MSNL probes from the same vendor.

Data analysis. All AFM images were analyzed using the Femtoscan Online software package (Advanced Technologies Center, Moscow, Russia), which enables precise tracing of the DNA molecules. Femtoscan software measures the contour length pixel-by-pixel automatically during tracing of the molecule by the mouse. The tracing was performed from the end of the DNA-protein complex (for convenience, from the short end of the complex) to the middle of the protein and then continued from that point to another end of the DNA molecule. The data obtained over an ensemble of the complexes are assembled as a table and the data analyzed using Origin software to generate the histogram. It was approximated with a Gaussian and the mean values and errors (SD and SEM) were calculated from this distribution. Fig. S7 illustrates a typical contour length distribution for bare DNA and contour length histograms for complexes are shown in Figs. 2 and 5.

The protein height and volumes r were measured with the cross-section option. The volume was calculated by *V* = 3.15 × *H*/6 × (0.75 × *D*_1_ × *D*_2_+*H*^2^), in which D_1_ and D_2_ are the diameters of the protein, which were measured two times, and H is the highest height of the two measurements of the protein^18^,^24^

## Supplementary Material

Supplementary Information Supplementary Information

## References

[b1] Kogoma T. Stable DNA replication: interplay between DNA replication, homologous recombination, and transcription. Microbiol Mol Biol Rev 61, 212–238 (1997). 9184011 10.1128/mmbr.61.2.212-238.1997 PMC232608

[b2] Kuzminov A. Recombinational repair of DNA damage in Escherichia coli and bacteriophage lambda. Microbiol Mol Biol Rev 63, 751–813 (1999). 10585965 10.1128/mmbr.63.4.751-813.1999 PMC98976

[b3] Kowalczykowski S. C. Initiation of genetic recombination and recombination-dependent replication. Trends Biochem Sci 25, 156–165 (2000). 10754547 10.1016/s0968-0004(00)01569-3

[b4] Bhattacharyya B. *et al.* Structural mechanisms of PriA-mediated DNA replication restart. Proc Natl Acad Sci U S A 111, 1373–1378 (2014). 24379377 10.1073/pnas.1318001111 PMC3910646

[b5] Cox M. M. *et al.* The importance of repairing stalled replication forks. Nature 404, 37–41 (2000). 10716434 10.1038/35003501

[b6] Cox M. M. Recombinational DNA repair of damaged replication forks in Escherichia coli: questions. Annu Rev Genet 35, 53–82 (2001). 11700277 10.1146/annurev.genet.35.102401.090016

[b7] Manosas M. *et al.* RecG and UvsW catalyse robust DNA rewinding critical for stalled DNA replication fork rescue. Nat Commun 4, 2368 (2013). 24013402 10.1038/ncomms3368 PMC3778716

[b8] Slocum S. L., Buss J. A., Kimura Y. & Bianco P. R. Characterization of the ATPase activity of the Escherichia coli RecG protein reveals that the preferred cofactor is negatively supercoiled DNA. J Mol Biol 367, 647–664 (2007). 17292398 10.1016/j.jmb.2007.01.007 PMC1913479

[b9] Buss J. A., Kimura Y. & Bianco P. R. RecG interacts directly with SSB: implications for stalled replication fork regression. Nucleic Acids Res 36 (2008). 10.1093/nar/gkn795 PMC2602778 18986999

[b10] Singleton M. R., Scaife S. & Wigley D. B. Structural analysis of DNA replication fork reversal by RecG. Cell 107, 79–89 (2001). 11595187 10.1016/s0092-8674(01)00501-3

[b11] Abd Wahab S., Choi M. & Bianco P. R. Characterization of the ATPase activity of RecG and RuvAB proteins on model fork structures reveals insight into stalled DNA replication fork repair. J Biol Chem 288, 26397–26409 (2013). 23893472 10.1074/jbc.M113.500223 PMC3772186

[b12] Hargreaves D., Rafferty J. B., Sedelnikova S. E., Lloyd R. G. & Rice D. W. Crystallization of Escherichia coli RuvA complexed with a synthetic Holliday junction. Acta Crystallogr D Biol Crystallogr 55, 263–265 (1999). 10089419 10.1107/S0907444998006672

[b13] Kur J., Olszewski M., Dlugolecka A. & Filipkowski P. Single-stranded DNA-binding proteins (SSBs) - sources and applications in molecular biology. Acta Biochim Pol 52, 569–574 (2005). 16082412

[b14] Lohman T. M. & Ferrari M. E. Escherichia coli single-stranded DNA-binding protein: multiple DNA-binding modes and cooperativities. Annu Rev Biochem 63, 527–570 (1994). 7979247 10.1146/annurev.bi.63.070194.002523

[b15] Shereda R. D., Kozlov A. G., Lohman T. M., Cox M. M. & Keck J. L. SSB as an organizer/mobilizer of genome maintenance complexes. Crit Rev Biochem and Mol Biol 43, 289–318 (2008). 18937104 10.1080/10409230802341296 PMC2583361

[b16] Bianco P. R. & Hurley E. M. The type I restriction endonuclease EcoR124I, couples ATP hydrolysis to bidirectional DNA translocation. J Mol Biol 352, 837–859 (2005). 16126220 10.1016/j.jmb.2005.07.055

[b17] McGlynn P. & Lloyd R. G. RecG helicase activity at three- and four-strand DNA structures. Nucleic Acids Res 27, 3049–3056 (1999). 10454599 10.1093/nar/27.15.3049 PMC148529

[b18] Shlyakhtenko L. S., Lushnikov A. Y., Miyagi A. & Lyubchenko Y. L. Specificity of binding of single-stranded DNA-binding protein to its target. Biochemistry 51, 1500–1509 (2012). 22304461 10.1021/bi201863z PMC3848610

[b19] McGlynn P. & Lloyd R. G. Genome stability and the processing of damaged replication forks by RecG. Trends Genet 18, 413–419 (2002). 12142010 10.1016/s0168-9525(02)02720-8

[b20] Buss J. A., Kimura Y. & Bianco P. R. RecG interacts directly with SSB: implications for stalled replication fork regression. Nucleic Acids Res 36, 7029–7042 (2008). 18986999 10.1093/nar/gkn795 PMC2602778

[b21] Lohman T. M., Green J. M. & Beyer R. S. Large-scale overproduction and rapid purification of the Escherichia coli ssb gene product. Expression of the ssb gene under lambda PL control. Biochemistry 25, 21–25 (1986). 3006753 10.1021/bi00349a004

[b22] Lohman T. M. & Overman L. B. Two binding modes in Escherichia coli single strand binding protein-single stranded DNA complexes. Modulation by NaCl concentration. J Biol Chem 260, 3594–3603 (1985). 3882711

[b23] Lyubchenko Y. L., Shlyakhtenko L. S. & Ando T. Imaging of nucleic acids with atomic force microscopy. Methods 54, 274–283 (2011). 21310240 10.1016/j.ymeth.2011.02.001 PMC3114274

[b24] Shlyakhtenko L. S. *et al.* Nanoscale structure and dynamics of ABOBEC3G complexes with single-stranded DNA. Biochemistry 51, 6432–6440 (2012). 22809226 10.1021/bi300733d PMC3448016

