# Supplementary material for: Remodeling of RecG Helicase at the DNA Replication Fork by SSB Protein
Source: Sci Rep. 2015 Apr 29;5:9625. doi: 10.1038/srep09625 (PMC4894433; doi:10.1038/srep09625)
Supplement: Supplementary Information [file srep09494-s1.doc]

**Remodeling of RecG Helicase at the DNA Replication Fork by SSB Protein**

Zhiqiang Sun^1^, Hui Yin Tan^2^, Piero R. Bianco^2^, Yuri L. Lyubchenko^1*^

^1^Department of Pharmaceutical Sciences, University of Nebraska Medical Center, Omaha, NE 68198-6025, USA

^2^Department of Microbiology and Immunology, University at Buffalo, SUNY, Buffalo, NY 14214, USA

**Supplementary Materials:**

Supplementary Fig. S1 to S7


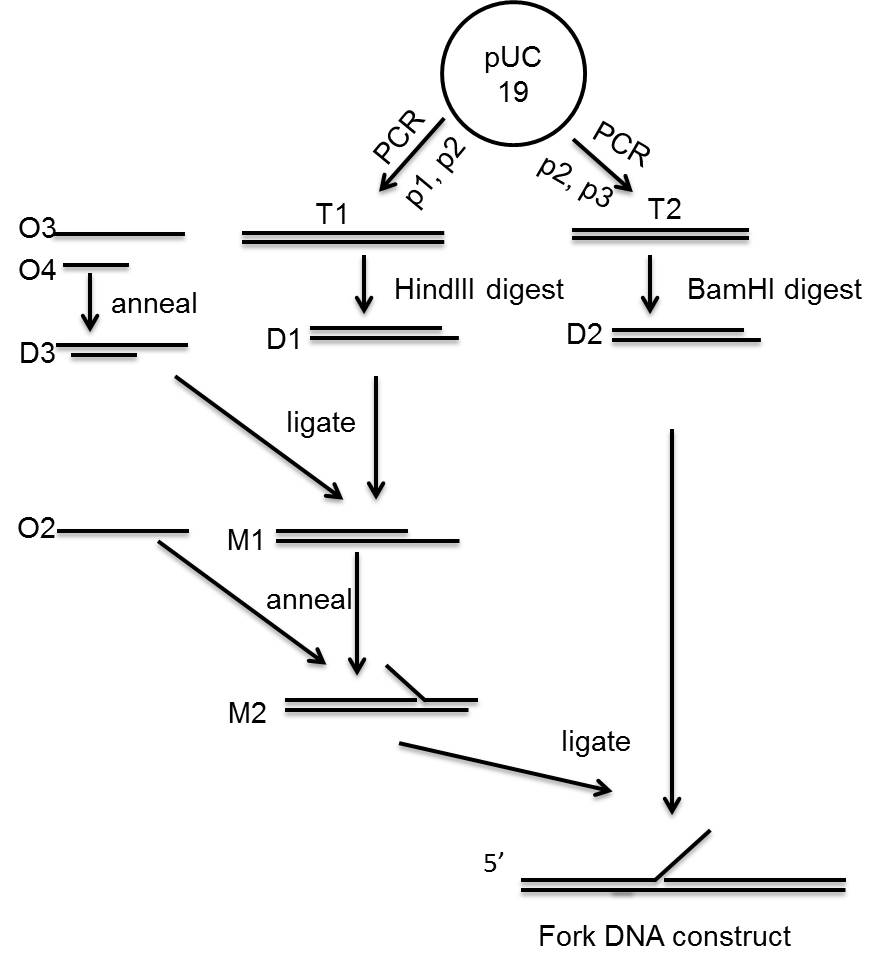


Supplementary Figure S1. The assembly of the fork DNA construct.


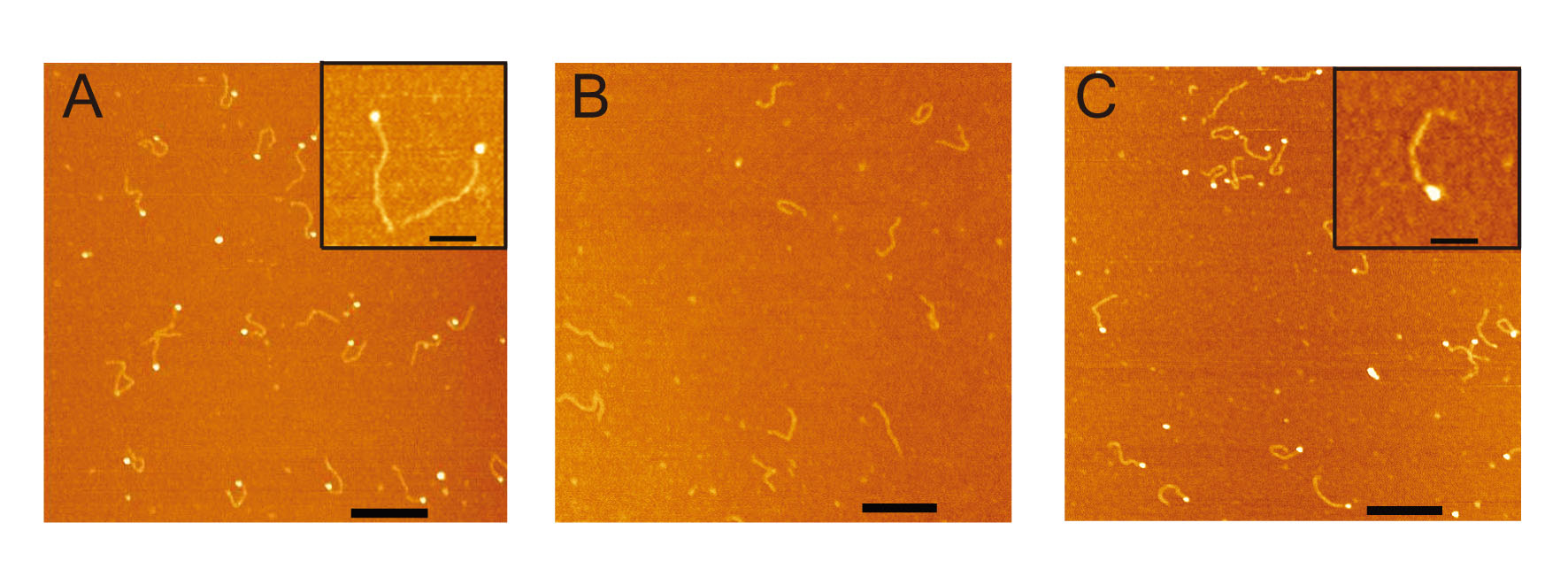


**Supplementary Figure S2. AFM images of protein-DNA complexes.** The fork substrate is replaced with a tailed-DNA, which contains the same 3’-ssDNA as in the fork design, but is now positioned at one end of the DNA duplex (tail DNA substrate). (A) Tail DNA mixed with SSB, (B) Tail DNA mixed with RecG, (C) Tail DNA mixed with SSB followed by RecG. Bar size 200 nm. Insets show enlarged images of complexes. Bar size 50 nm.

**
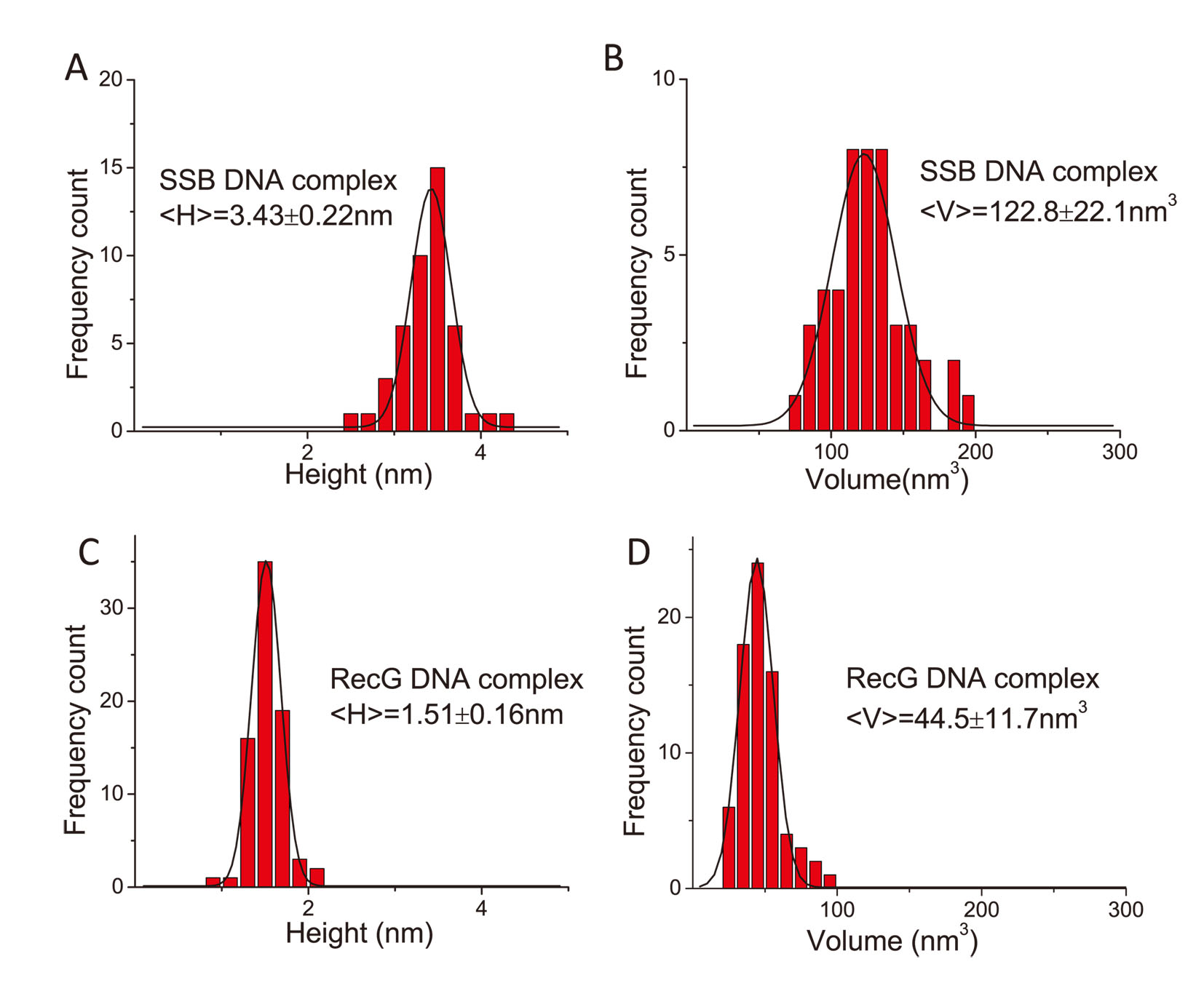
**

**Supplementary Figure S3**. The height and volume measurements for SSB are shown in (A, B) (n=47), and for RecG in (C, D) (n=74), when complexed with DNA. The histograms were fitted by Gaussians. The mean values ± SD are indicated on the histograms.

**
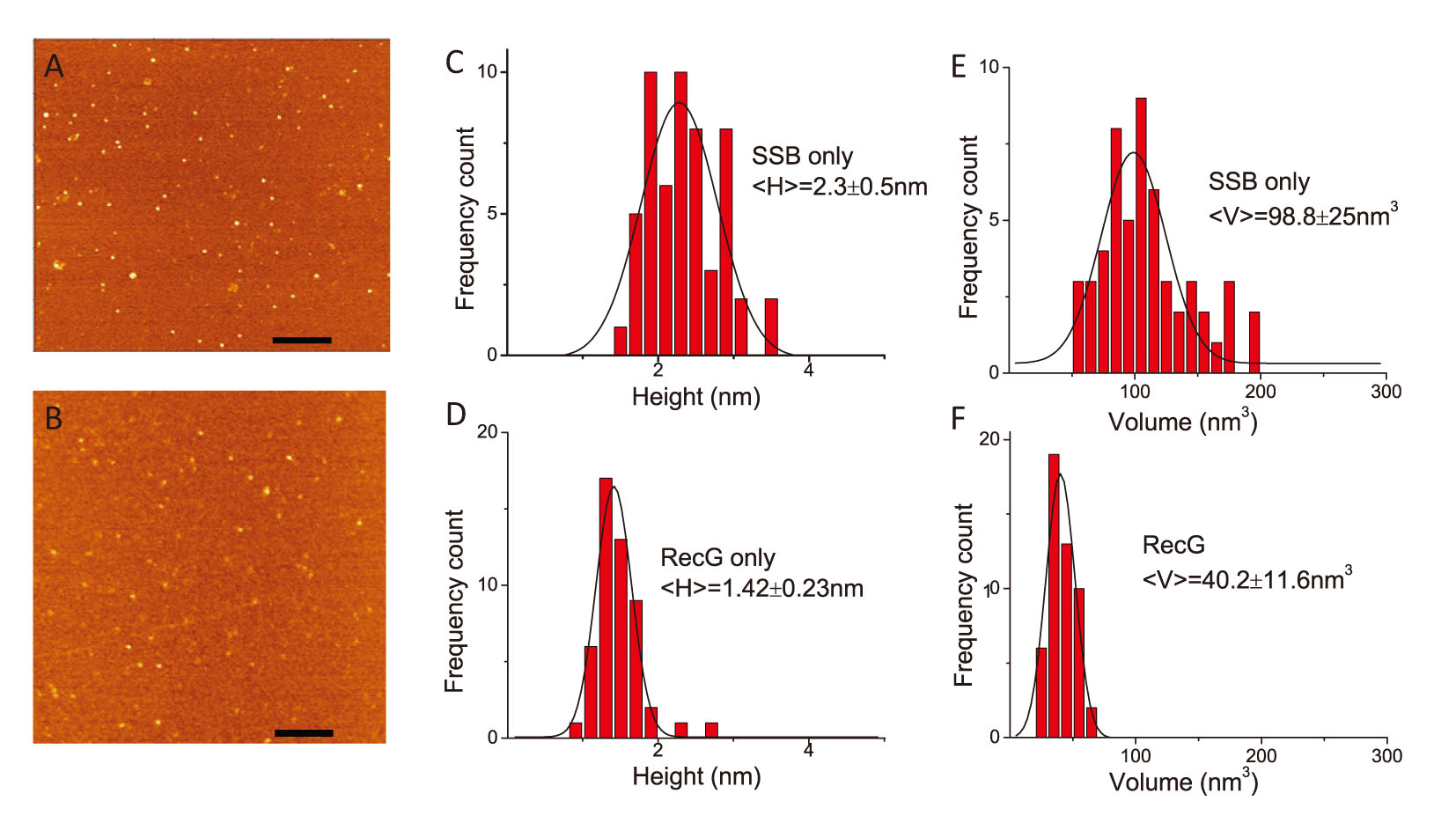
**

**Supplementary Figure S4.** AFM data of free SSB and RecG. (A) and (B) show AFM images of free SSB and RecG, respectively, when deposited onto the APS mica surface. The height and volume distributions for SSB are shown in (C, E) (n=54), and for RecG in (D, F) (n=50). The histograms were fitted by Gaussians. The mean values ± SD are indicated on the histograms. Bar size 200nm.

**
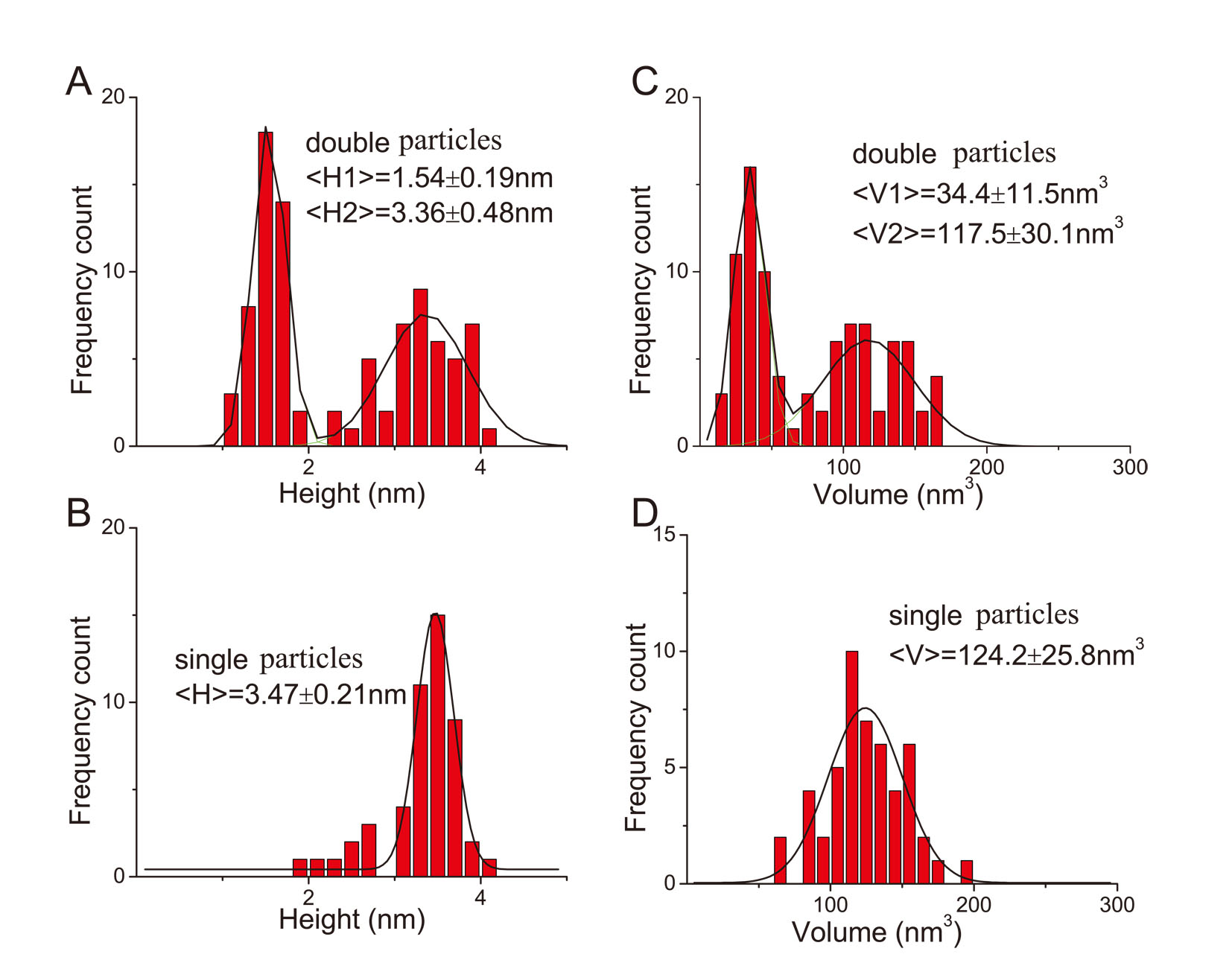
**

**Supplementary Figure S5**. The height and volume distributions for the double-particle complexes (A, C) (n=42) and single-particle complexes (B, D) (n=51). In these experiments, DNA was mixed with SSB first, and then RecG was added to the complex (setup 1). The mean values ± SD are indicated on the histograms.

**
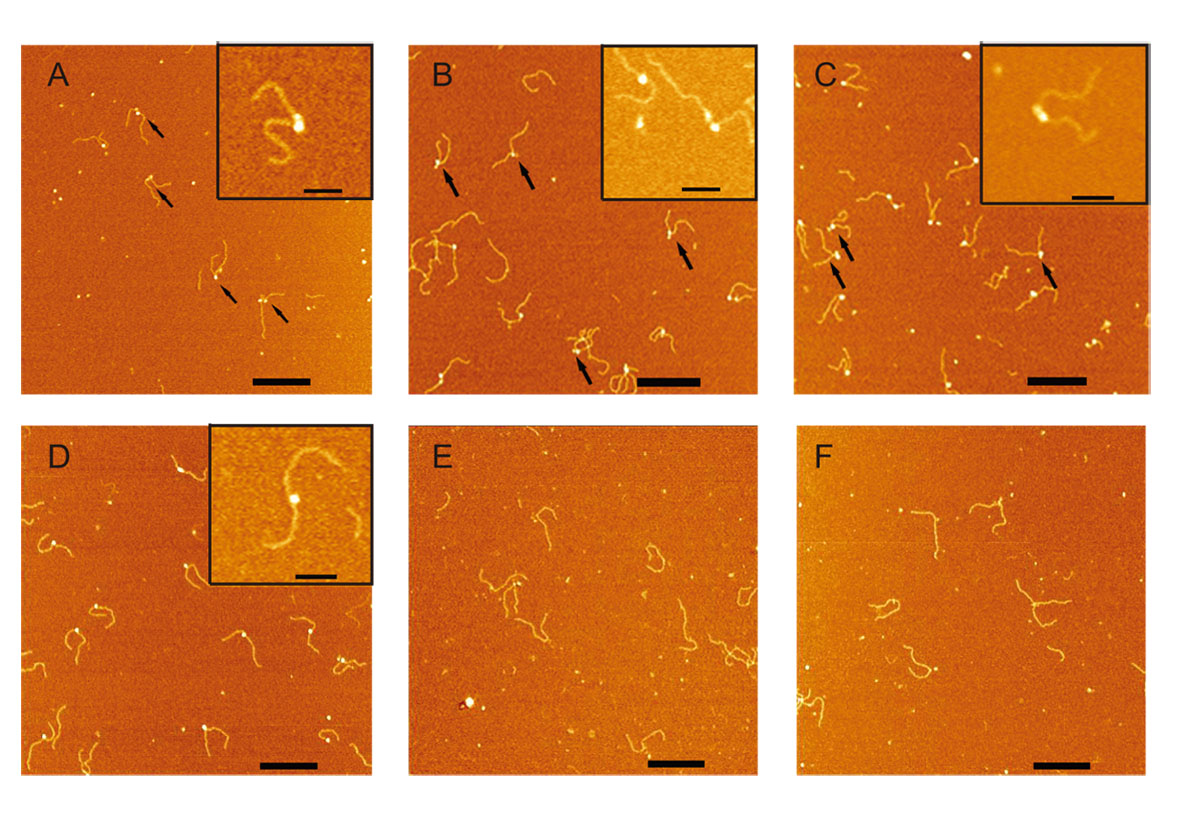
**

**Supplementary Figure S6.** **Typical AFM images of complexes on the fork DNA with different proteins and different buffer compositions**. (A) DNA is mixed with SSB first, then RecG is added to the complexes. The black arrows show the double particles. (B) SSB and RecG are premixed and then incubated with DNA. (C) DNA is mixed with SSB first, then RecG is added; the buffer contains 1 mM ATP. (D) The same as in (C) but SSBΔC is used. (E) DNA is mixed with gp32 for 10 min. (F) DNA is mixed with gp32 first, then RecG is added. Insets show enlarged images of complexes. Bar size 50 nm.


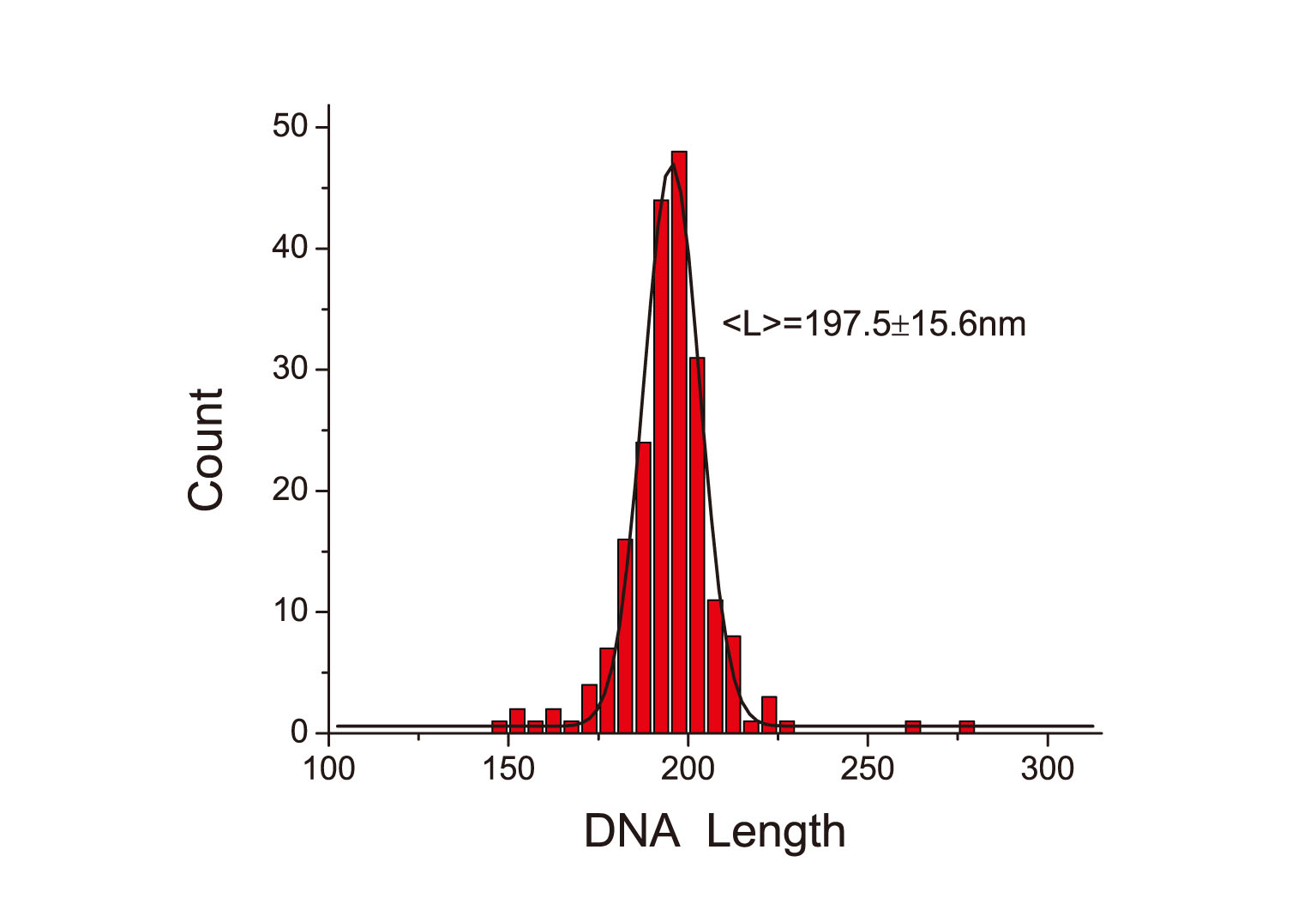


Figure S7: The length distribution for free fork DNA. The histogram was fitted with a Gaussian and the mean values ± SD are indicated are shown in the figure.
